# Supplementary material for: Non-resonant light scattering in dispersions of 2D nanosheets
Source: Nat Commun. 2018 Nov 1;9:4553. doi: 10.1038/s41467-018-07005-3 (PMC6212482; doi:10.1038/s41467-018-07005-3)
Supplement: Supplementary file 1 — Supplementary Information [file 41467_2018_7005_MOESM1_ESM.pdf]

**Supplementary Information for**

# Non-resonant light scattering in dispersions of 2D nanosheets

Andrew Harvey,[1,2] Claudia Backes,[3] John B. Boland,[1,2] Xiaoyun He,[1,2] Aideen Griffin, [1,2] Beata Szydlowska,[1,2] Cian Gabbett,[1,2] John F. Donegan[1,2] and Jonathan N. Coleman[1,2]*

[1]*CRANN & AMBER, Trinity College Dublin, Dublin 2, Ireland*

[2]*School of Physics, Trinity College Dublin, Dublin 2, Ireland*

[3] *Chair of Applied Physical Chemistry, University of Heidelberg, Im Neuenheimer Feld 253, 69120 Heidelberg, Germany*

*colemaj@tcd.ie

**Content**

*Supplementary Figures*:

- Raman spectra
- AFM
- Extinction/Absorbance/Scattering coefficient spectra
- Concentration dependence of scattering spectra
- Scattering exponent in water-surfactant and isopropanol
- Exemplary fits of scattering spectra
- Scattering exponent as function of various nanosheet dimensions
- Scattering from nanospheres
- Aggregation kinetics data

*Supplementary Tables*:

- Optical constants

*Supplementary Notes*:

- Discussion of Raman spectra
- Scattering parameter conversion and approximation for large disks
- Fitting of scattering exponent versus nanosheet dimension
- Scattering metrics for size estimation

*Supplementary References*

## Supplementary Figures

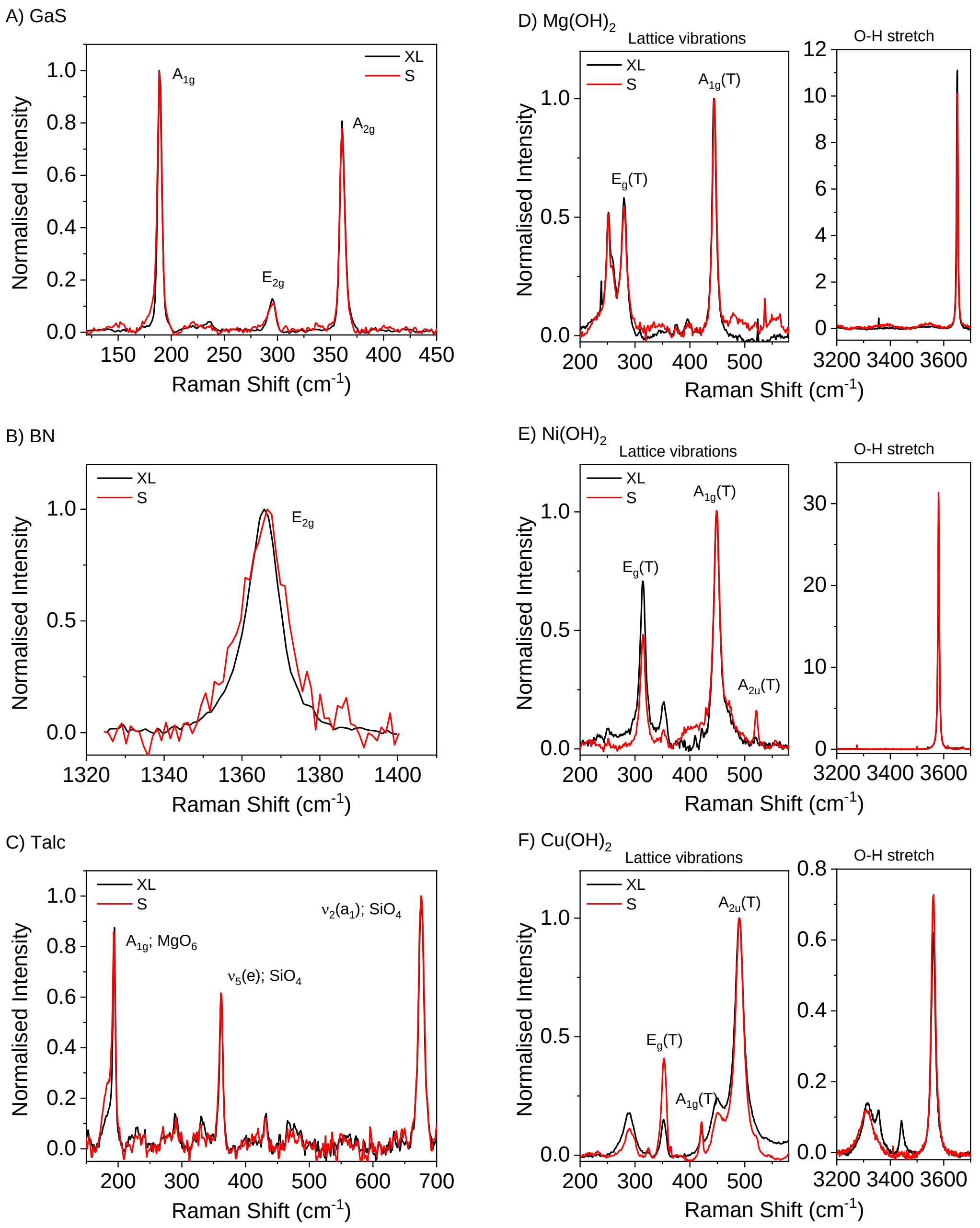


**Supplementary Figure 1**: **Raman spectroscopy**. Baseline corrected and normalised Raman spectra (532 nm excitation) of A) GaS, B) BN, C) Talc, D) $Mg(OH)_2$, E) $Ni(OH)_2$, F) $CU(OH)_2$ nanosheet films. Data from XL and S samples are shown in each case.

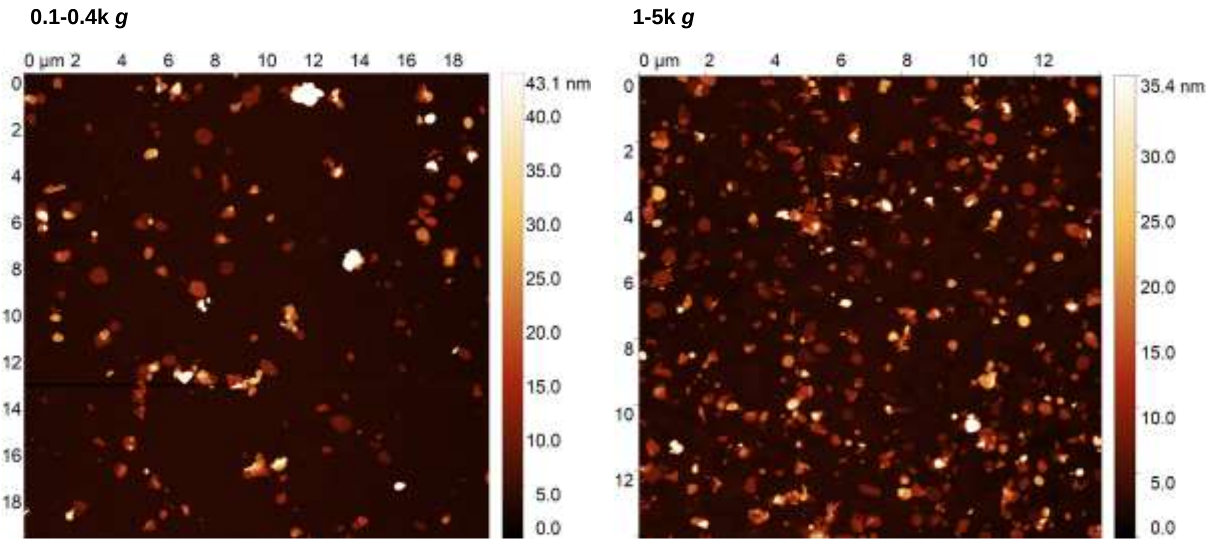


**Supplementary Figure 2: Examples of wide view AFM images of BN**.

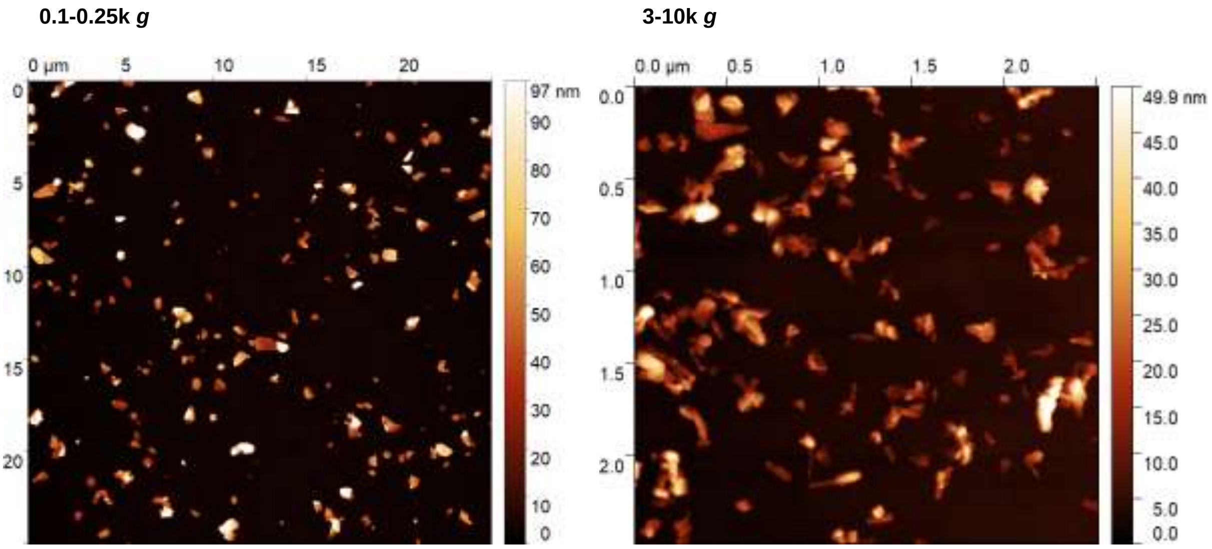


**Supplementary Figure 3: Examples of wide view AFM images of GaS**.

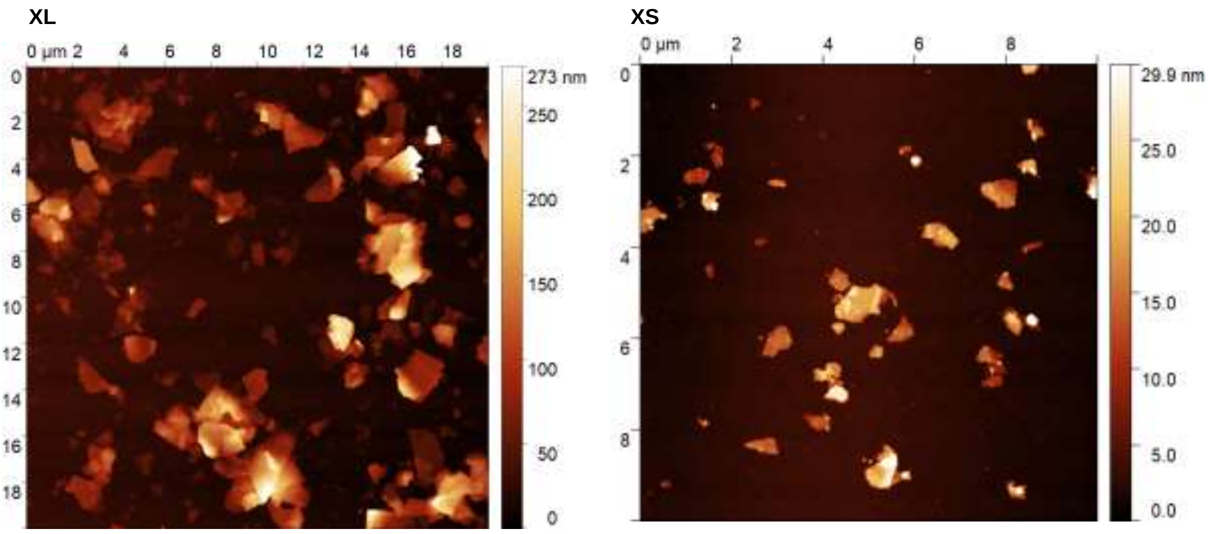


**Supplementary Figure 4: Examples of wide view AFM images of Talc.**

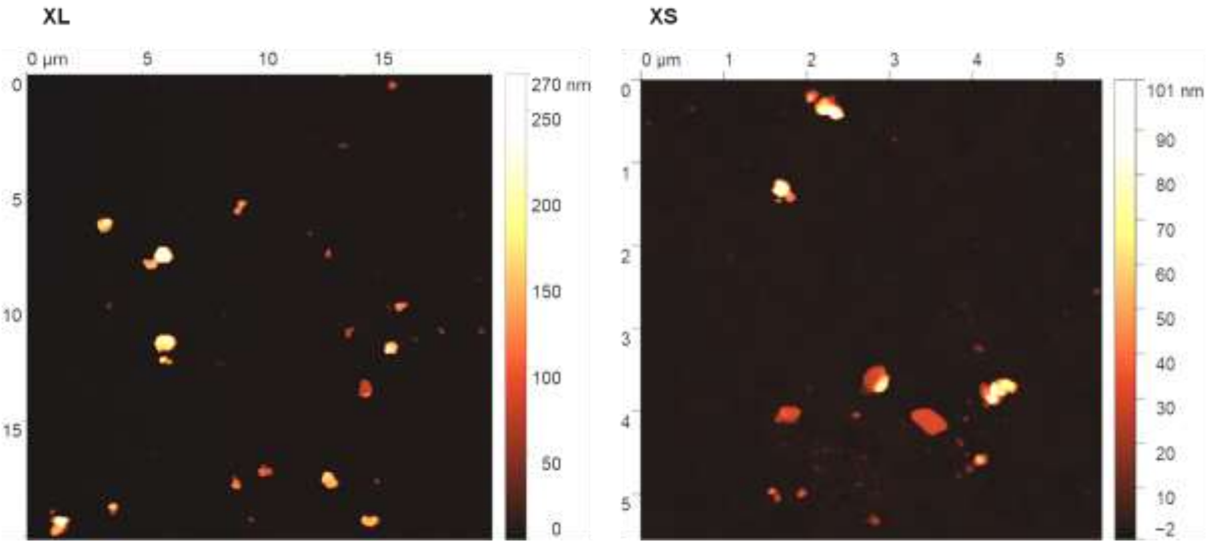


**Supplementary Figure 5: Examples of wide view AFM images of $Mg(OH)_2$.**

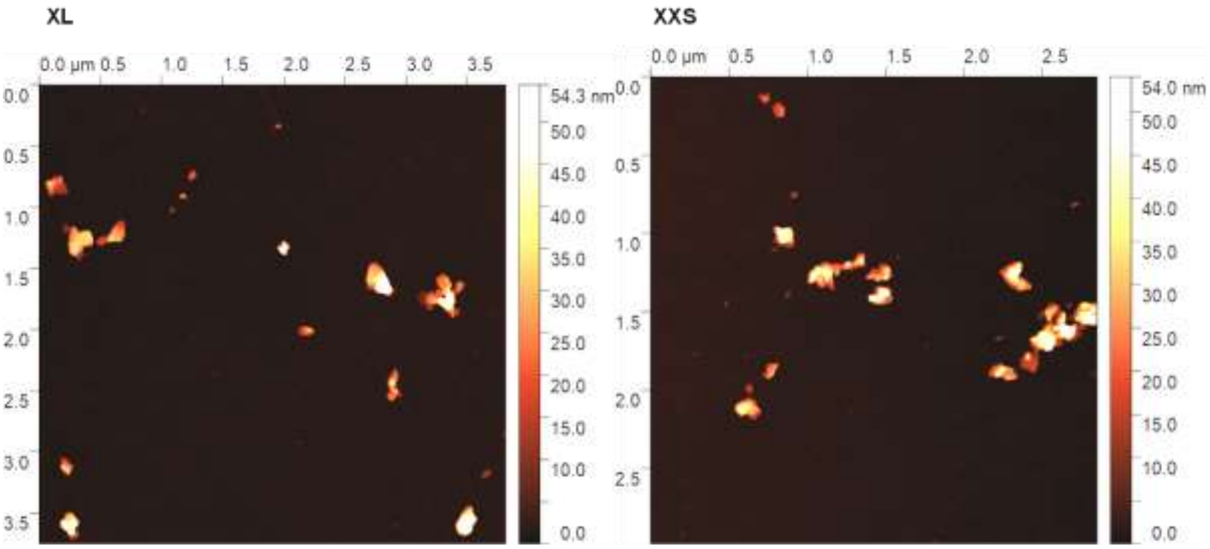


**Supplementary Figure 6: Examples of wide view AFM images of $Ni(OH)_2$.**

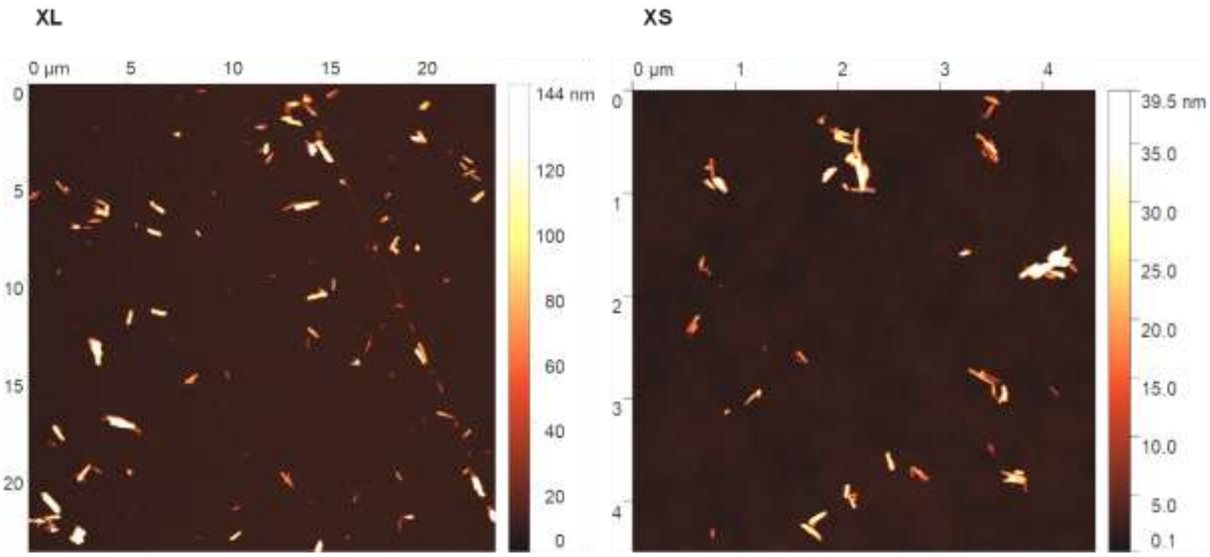


**Supplementary Figure 7: Examples of wide view AFM images of $Cu(OH)_2$.**

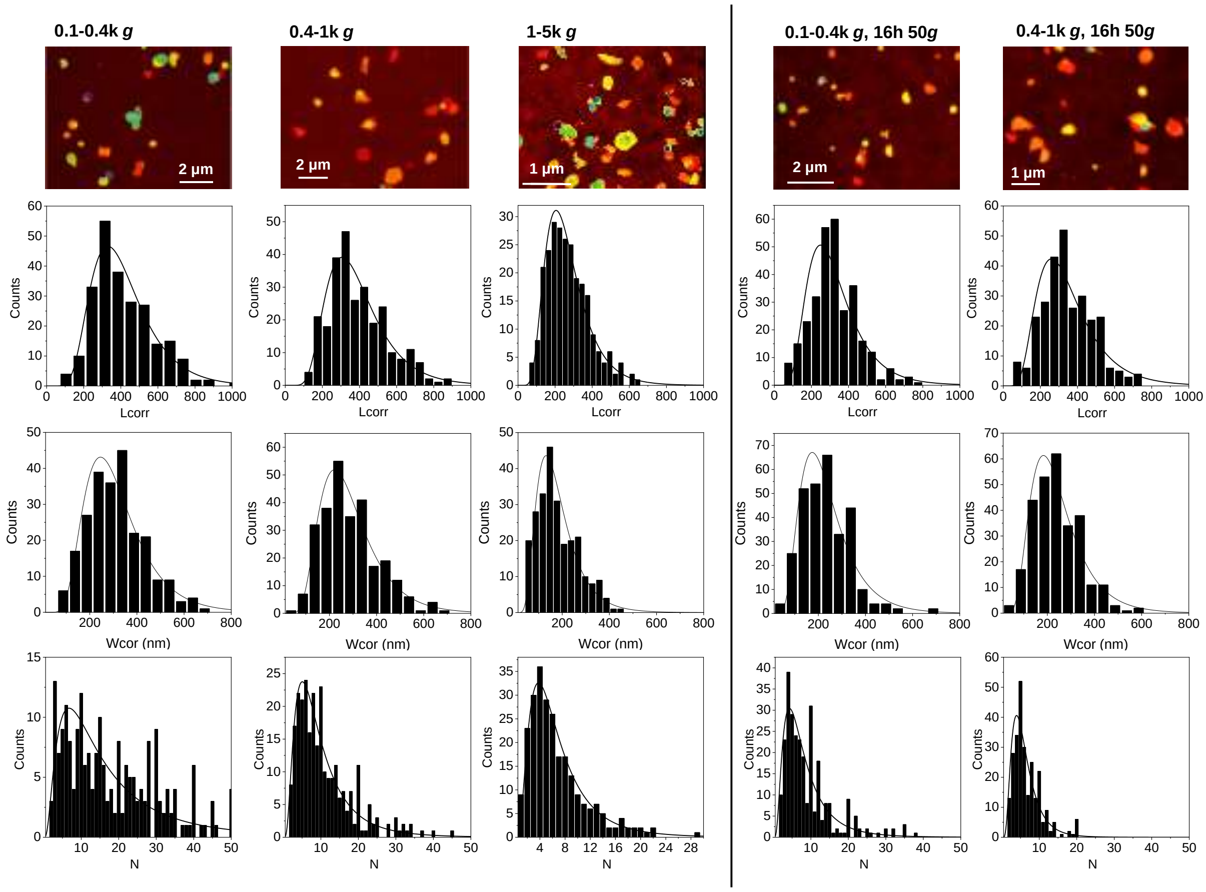


**Supplementary Figure 8**: **AFM images and histograms of BN**. Representative images (top row), length (row 2), width (row 3) and layer number histograms of the BN samples that were included in the study. The BN samples were prepared in analogy to the reported procedure.[1] It should be noted that our previous work had the target to establish layer number metrics. For this purpose, it was essential to prepare as thin nanosheet as possible by centrifuging at relatively high RCF (74000 *g*). However, dispersions produced in such a way showed negligible scattering and were thus excluded from the current work. Instead, the two largest samples were centrifuged at low RCF (50 *g*) overnight in an attempt to decouple the well-defined relationship between length and thickness obtained from standard cascades.[2]

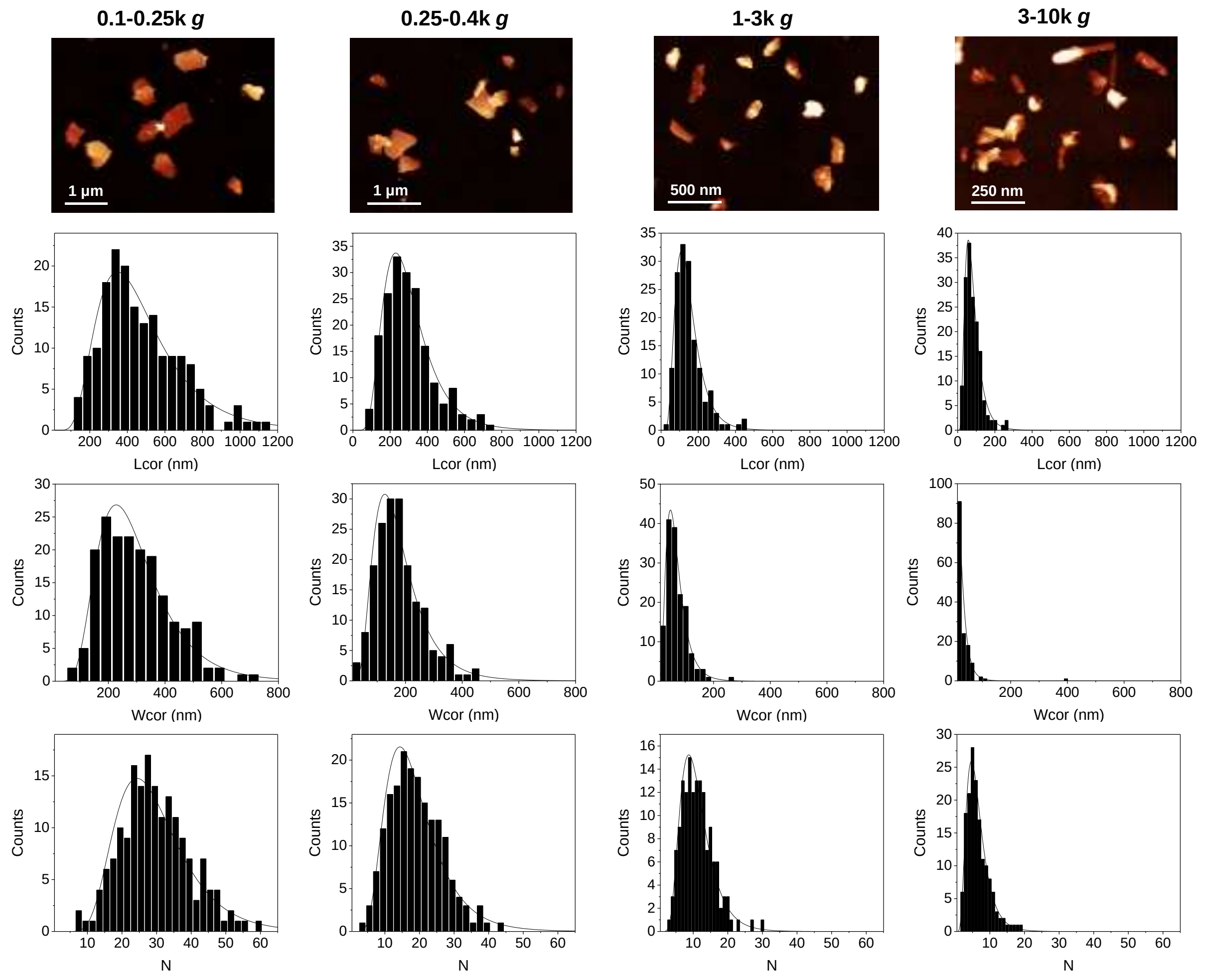


**Supplementary Figure 9**: **AFM images and histograms of GaS produced in a standard cascade**. Representative images (top row), length (row 2), width (row 3) and layer number histograms of the GaS produced in a standard cascade.

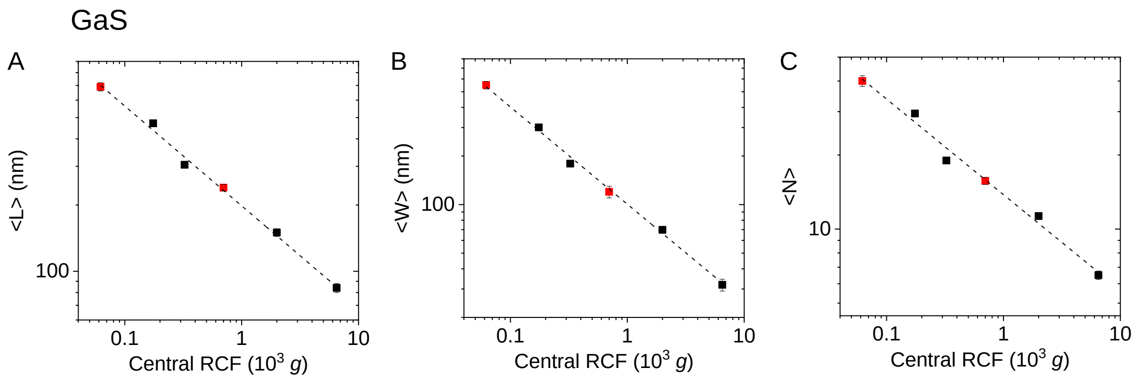


**Supplementary Figure 10: Scaling of GaS dimensions with central centrifugal acceleration**. A) Mean nanosheet length, B) Mean nanosheet width and C) Mean nanosheet layer number of GaS. The black data points were measured, the red data points obtained from the well-defined scaling by interpolation/extrapolation.

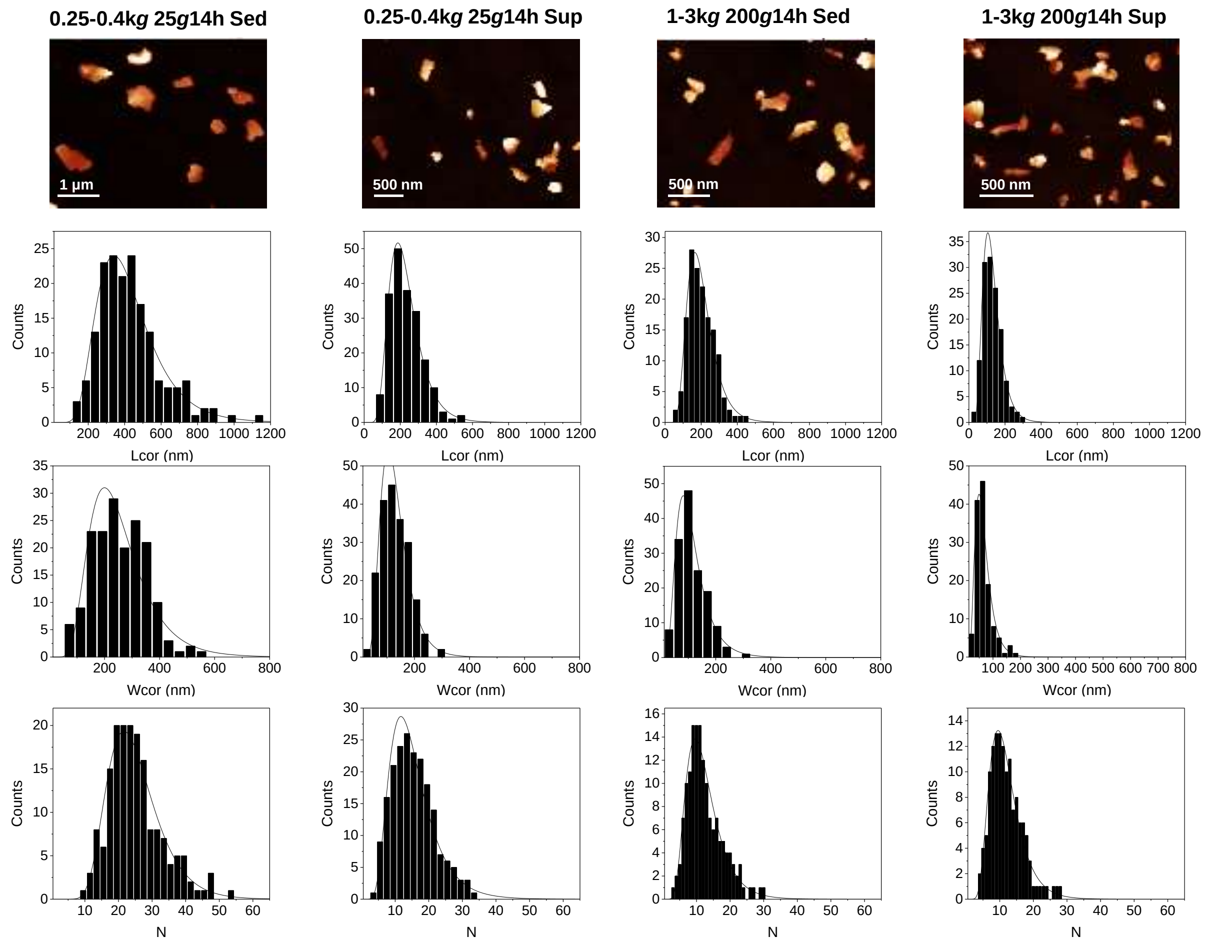


**Supplementary Figure 11**: **AFM images and histograms of GaS produced after overnight centrifugation.** Similar to BN, a second GaS sample set was prepared by subjecting two of the standard samples to overnight centrifugation to decouple the aspect ratio relationships. Specifically, we chose the samples that were initially trapped between 250-400 *g* and 1000-3000 *g* and centrifuged them for 14 h at 25 *g* and 200 *g*, respectively (i.e. significantly below the initial lower boundary). Both supernatants (S) and sediments (Sed) were subjected to AFM statistics (figure S5). Figure S6 shows a plot of different nanosheet dimensions versus each other confirming the decoupling worked at least to some extent. The figures shows representative images (top row), length (row 2), width (row 3) and layer number histograms of the GaS produced after centrifuging two of the standard samples overnight at low RCF.

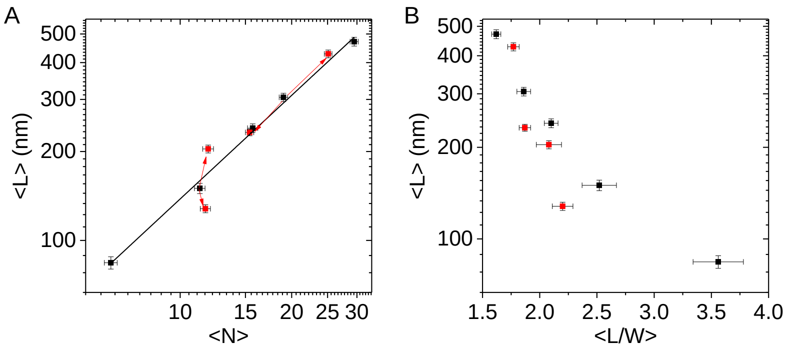


**Supplementary Figure 12: Plots of GaS nanosheet dimensions versus each other**. Samples from the standard centrifugation cascade (black data) are compared to the samples obtained after overnight centrifugation (both supernatant and sediment, red data). Plot of length as function of layer number shown a decoupling of the <L>-<N> relationship. B) Plot of mean nanosheet length as function of length-width aspect ratio.

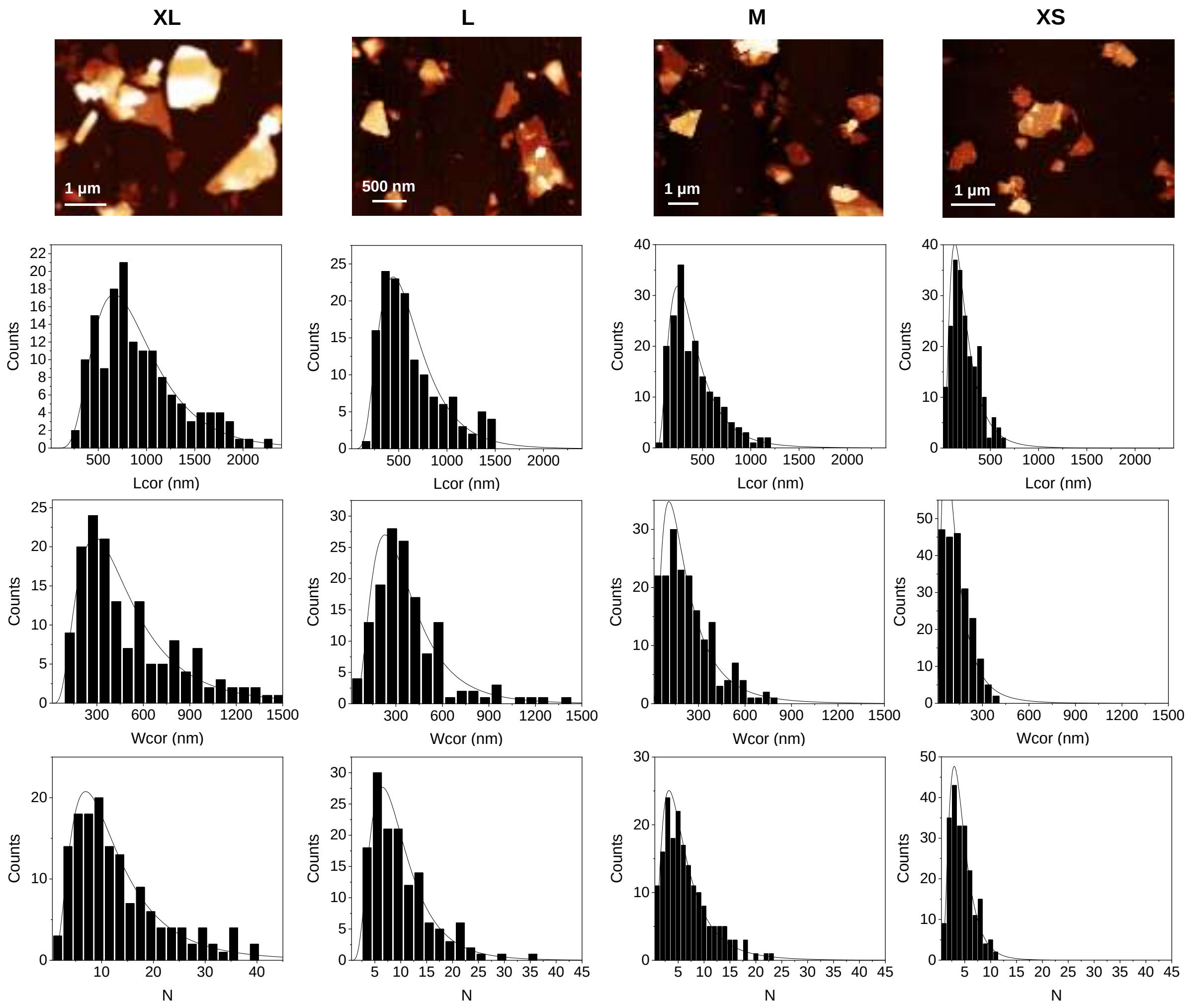


**Supplementary Figure 13**: **AFM images and histograms of Talc.** Representative images (top row), length (row 2), width (row 3) and layer number histograms.

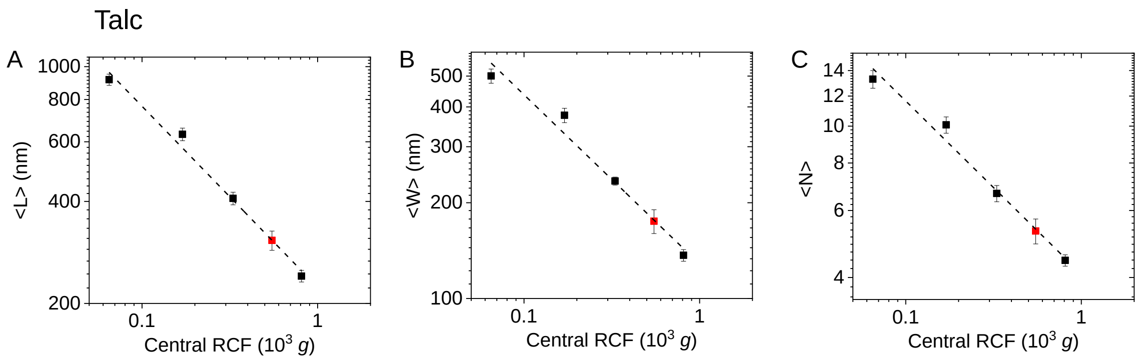


**Supplementary Figure 14: Scaling of Talc dimensions with central centrifugal acceleration**. A) Mean nanosheet length, B) Mean nanosheet width and C) Mean nanosheet layer number of talc. The black data points were measured, the red data point obtained from the well-defined scaling by interpolation.

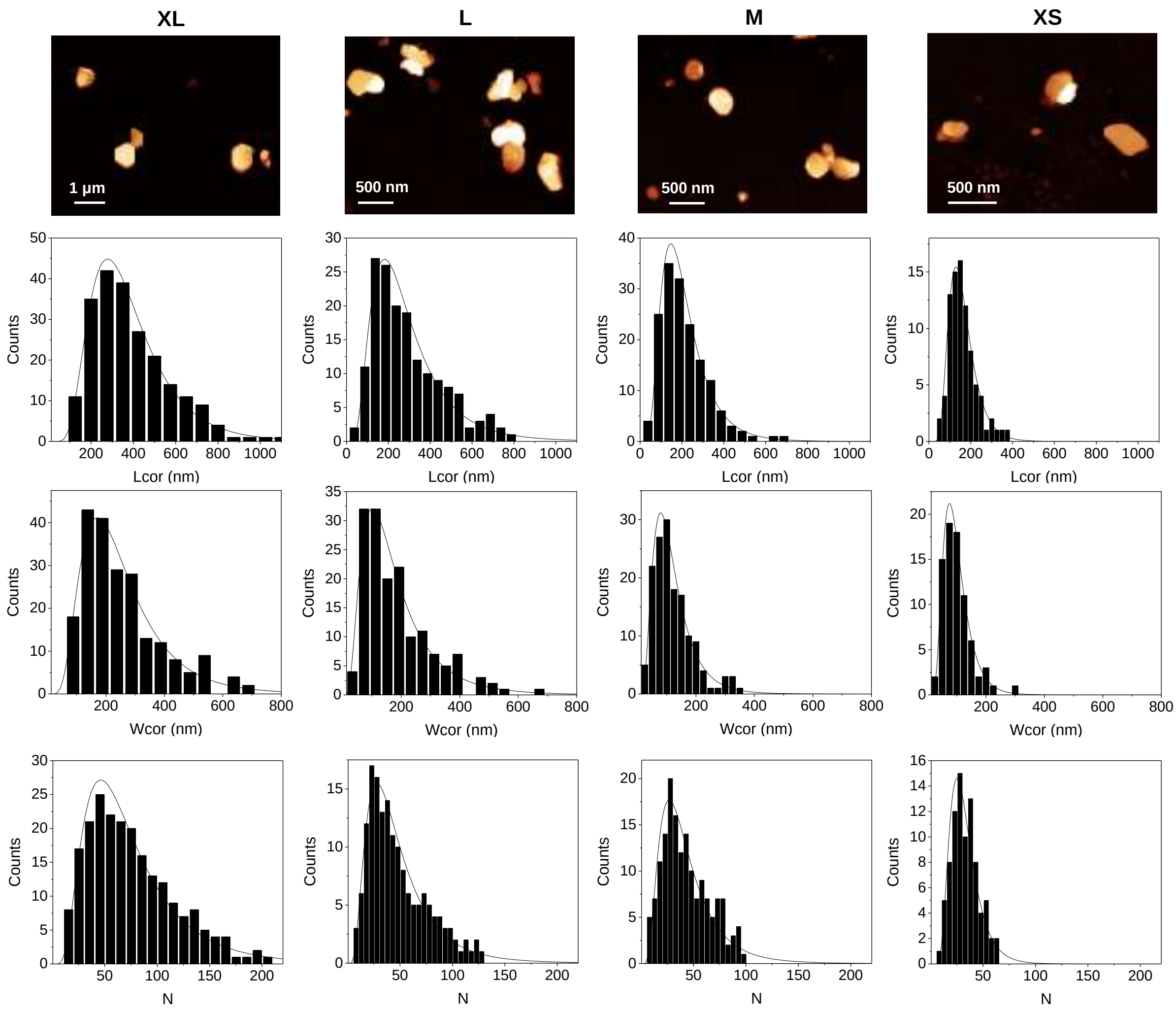


**Supplementary Figure 15**: **AFM images and histograms of Mg(OH)2**. Representative images (top row), length (row 2), width (row 3) and layer number histograms.

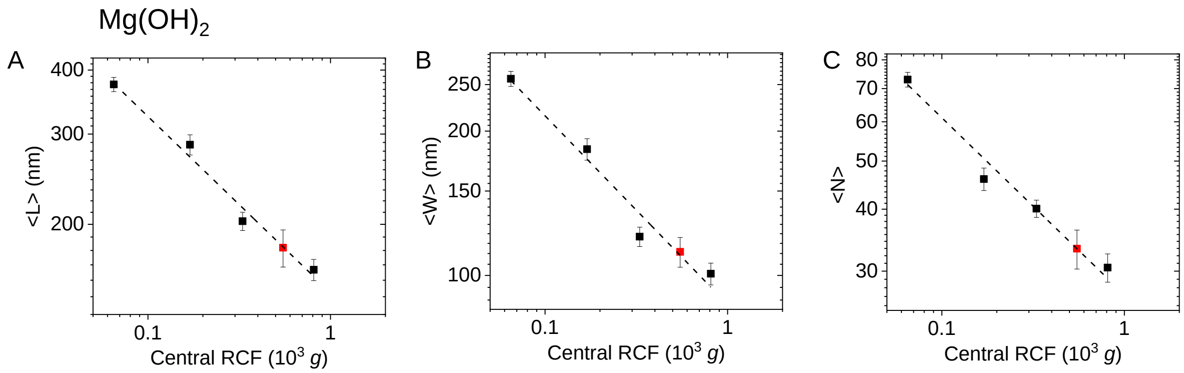


**Supplementary Figure 16: Scaling of Mg(OH)2 dimensions with central centrifugal acceleration**. A) Mean nanosheet length, B) Mean nanosheet width and C) Mean nanosheet layer number of Mg(OH)2. The black data points were measured, the red data point obtained from the well-defined scaling by interpolation.

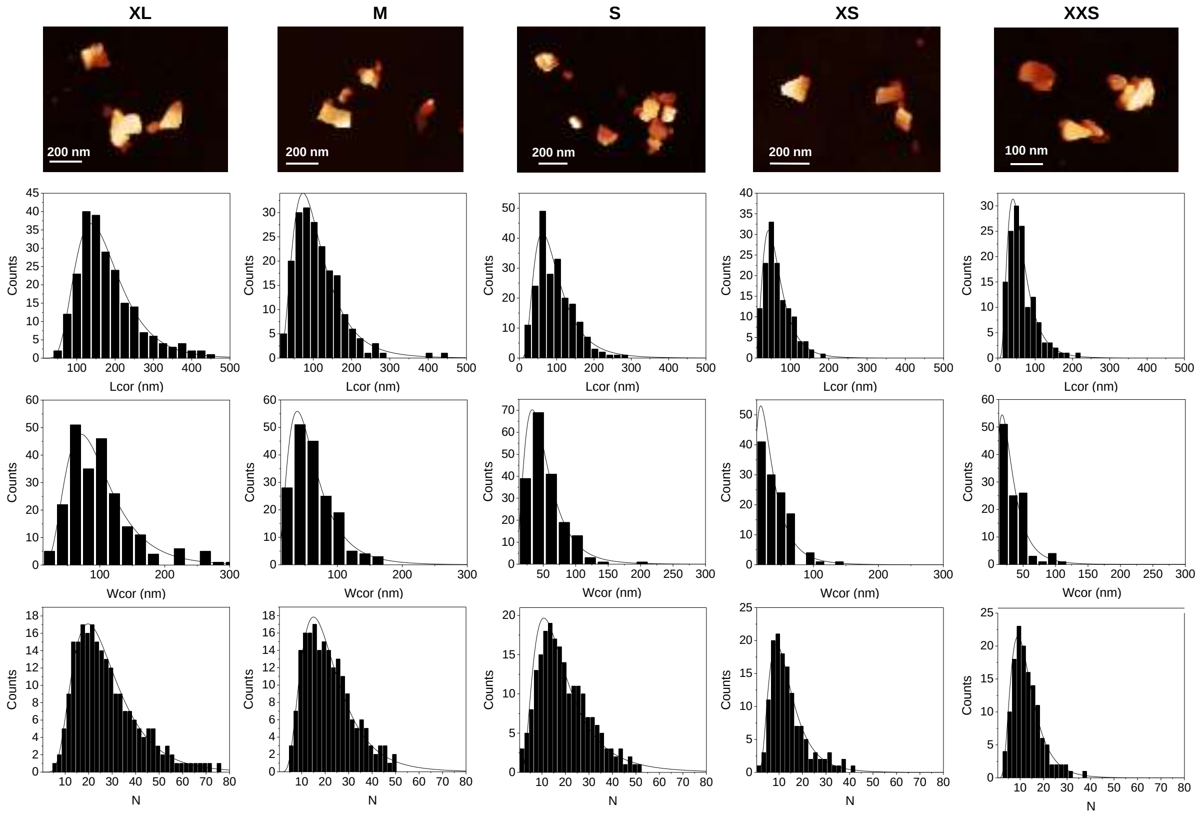


**Supplementary Figure 17**: **AFM images and histograms of Ni(OH)2.** Representative images (top row), length (row 2), width (row 3) and layer number histograms.

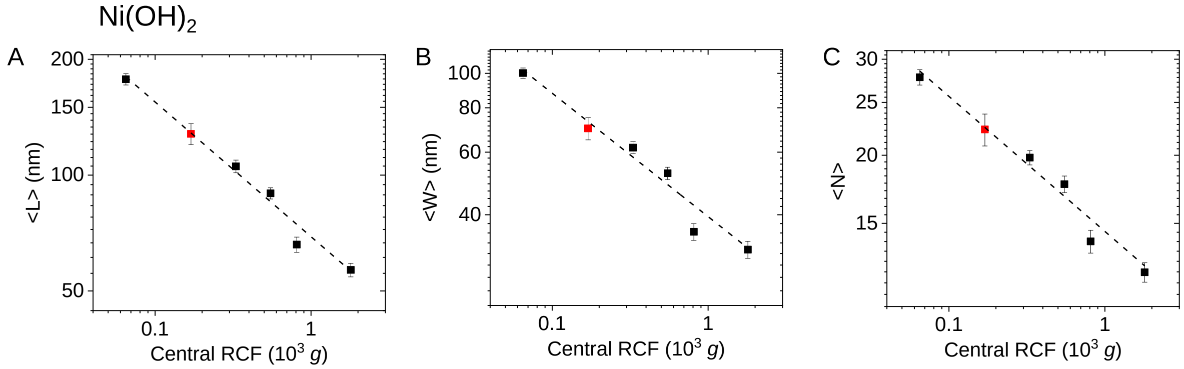


**Supplementary Figure 18: Scaling of Ni(OH)2 dimensions with central centrifugal acceleration**. A) Mean nanosheet length, B) Mean nanosheet width and C) Mean nanosheet layer number of Ni(OH)2. The black data points were measured, the red data point obtained from the well-defined scaling by interpolation.

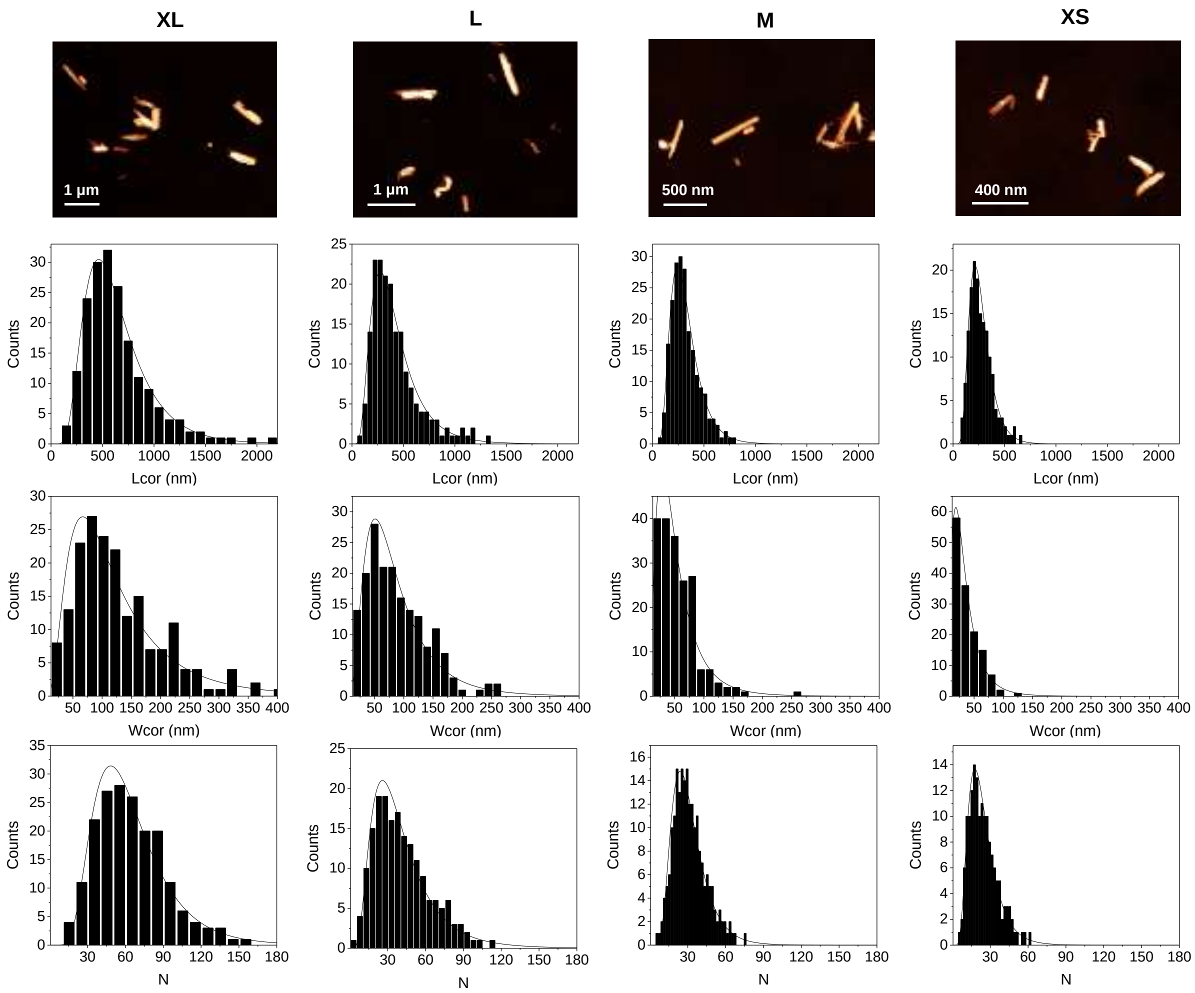


**Supplementary Figure 19**: **AFM images and histograms of Cu(OH)2.** Representative images (top row), length (row 2), width (row 3) and layer number histograms.

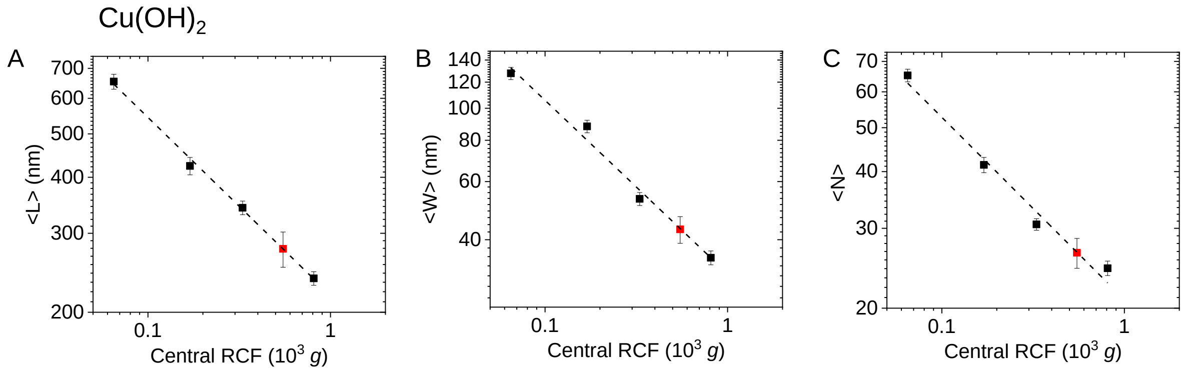


**Supplementary Figure 20: Scaling of Cu(OH)2 dimensions with central centrifugal acceleration**. A) Mean nanosheet length, B) Mean nanosheet width and C) Mean nanosheet layer number of Ni(OH)2. The black data points were measured, the red data point obtained from the well-defined scaling by interpolation.

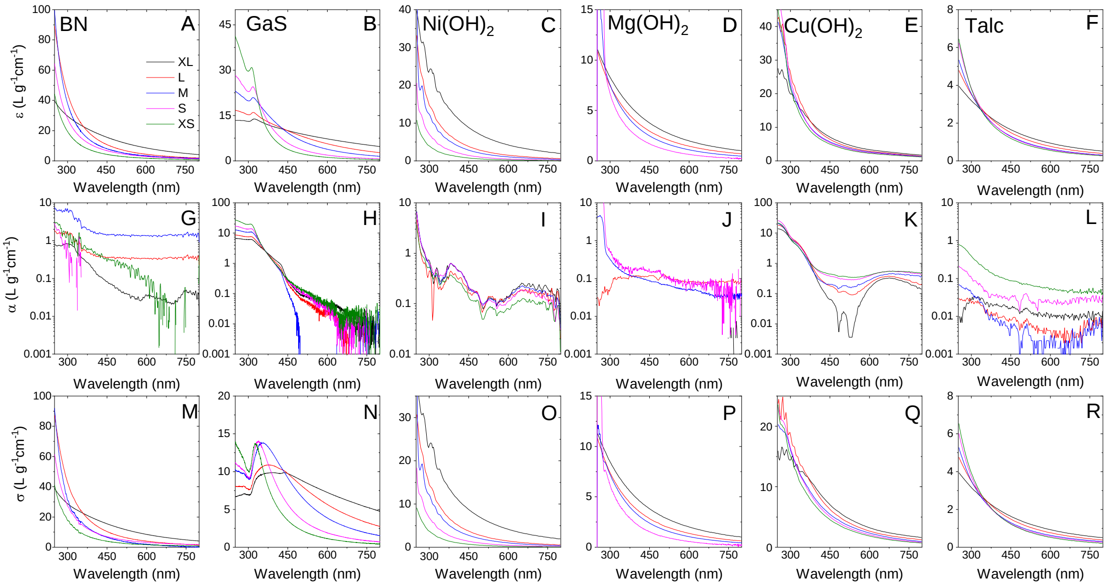


**Supplementary Figure 21: Extinction/Absorbance/Scattering spectra.** Extinction (A-E), absorption (F-J) and scattering (K-O) coefficient spectra for all size fractions (XL – XS) of the six chosen materials (BN, GaS, $Ni(OH)_2$, $Mg(OH)_2$, $Cu(OH)_2$ and Talc). Legend in A) applies to all panels. To obtain the scattering spectra of each fraction of each material (BN, GaS, $Ni(OH)_2$, $Mg(OH)_2$, $Cu(OH)_2$ and Talc) the extinction and absorption spectra were measured. These were then converted to coefficient spectra using the Beer Lambert law where Ext = εcl, Abs = αcl, Sca = σcl, with ε, α and σ being the extinction, absorption and scattering coefficients respectively. Nanosheet concentrations in all dispersions were obtained gravimetrically after filtering a known volume of the dispersion through alumina membranes and washing with ~ 500 mL deionised water to remove surfactant. For all materials the extinction (A-E) and scattering coefficient spectra (K-O) are mostly identical between 400 and 900 nm, displaying a power law behaviour scaling as $\lambda^{-n}$. This is attributed to the fact that all materials are wide bandgap semiconductors/insulators with no appreciable absorbance in this region. To illustrate this more clearly, the absorbance coefficient spectra are plotted on a semi-log scale. Therefore, the loss of transmitted light in the extinction spectra is exclusively due to non-resonant scattering..

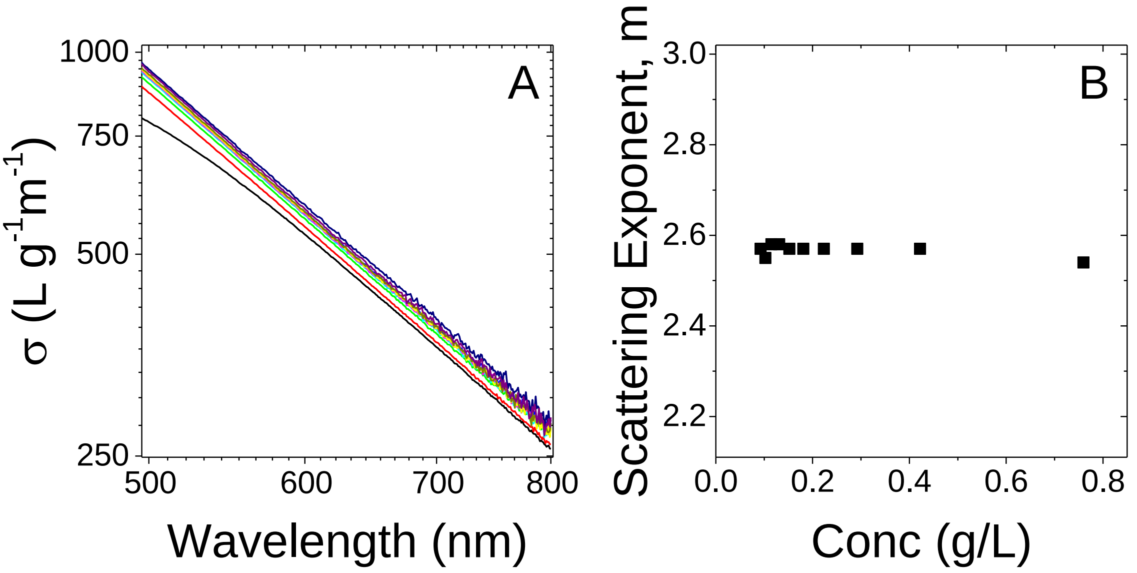


**Supplementary Figure 22: Concentration dependence.** To demonstrate that no multiple scattering events affect the data in the measured concentration regime (below optical densities of 0.5 at the maximum extinction), a high concentration BN sample (trapped between 400-1000 *g*) was diluted, scattering spectra measured and converted to scattering coefficient spectra. A) Scattering coefficient spectra plotted versus wavelength for differing concentrations of the BN sampled obtained by trapping between 400-1000 *g*. B) Scattering exponent (m) plotted against the concentration of the samples. The extracted scattering exponent does not change with nanosheet concentration confirming that the measurements are within the single scattering regime.

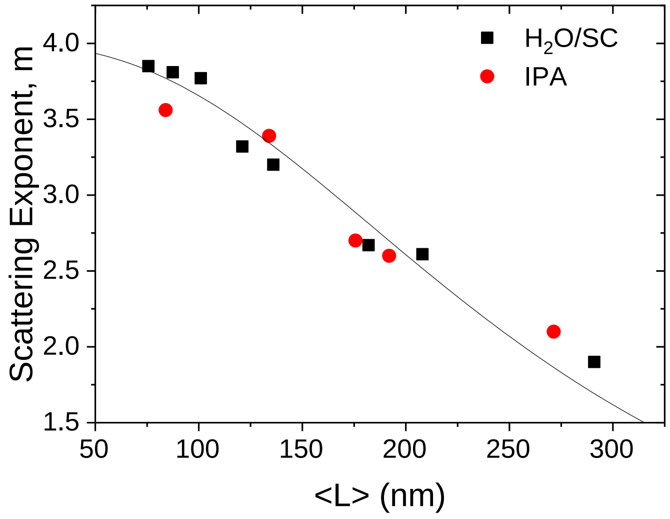


**Supplementary Figure 23: Impact of medium on scattering exponent.** Since the long wavelength regime is fitted to extract the scattering prefactor and exponent, no contribution from surfactant is expected. To test this, a sample set of Ni(OH)2 was prepared in isopropanol, the nanosheet size determined by TEM and the scattering exponent extracted from the optical spectra. This is plotted versus the nanosheet length together with the data obtained from the water-surfactant dispersion. As expected, the data falls on the same curve.

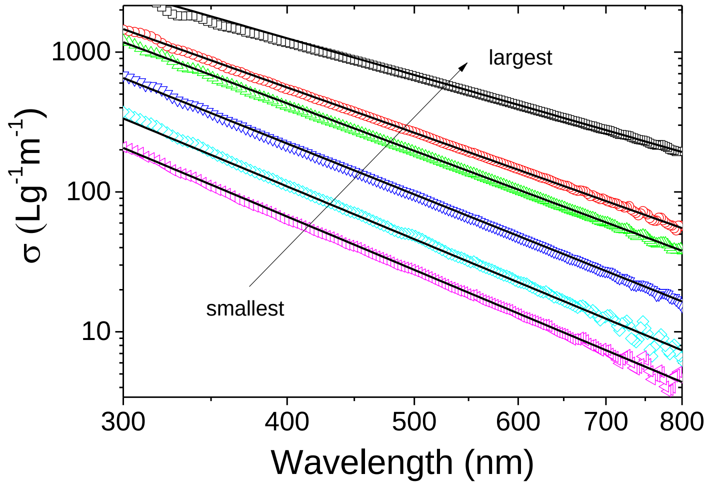


**Supplementary Figure 24: Fitting scattering spectra.** Examples of power law fits to the size-selected $Ni(OH)_2$ samples (open symbols data, lines fits). When the equation $\sigma = K\lambda^{-m}$ is fitted to these regions it is possible to obtain the scattering prefactor (K) and the scattering exponent (m).

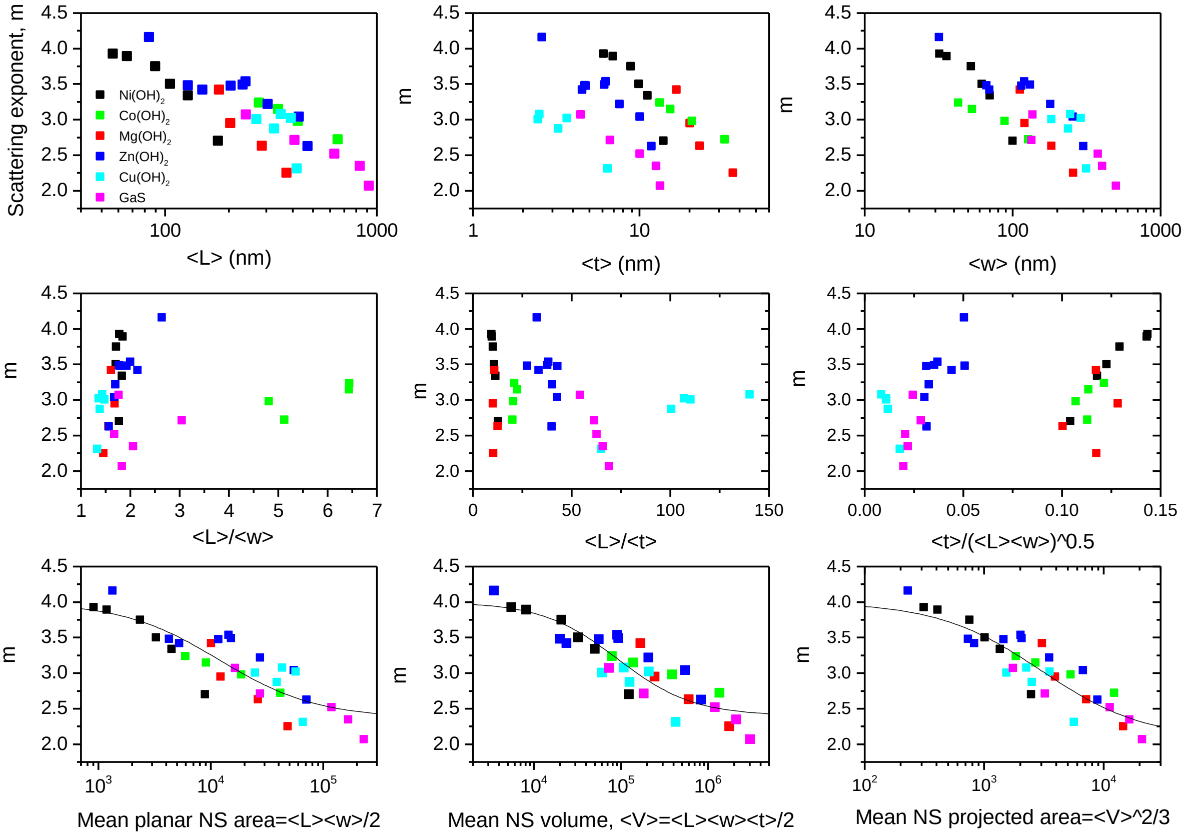


**Supplementary Figure 25**: **Identifying the *m*-mastercurve**. Plots of the scattering exponent versus various nanosheet dimensions. From these plots, mastercurve-like behaviour can be inferred for mean planar nanosheet area, volume and projected area (bottom row). To identify the best candidate, the data was fit as described in supplementary note 3.

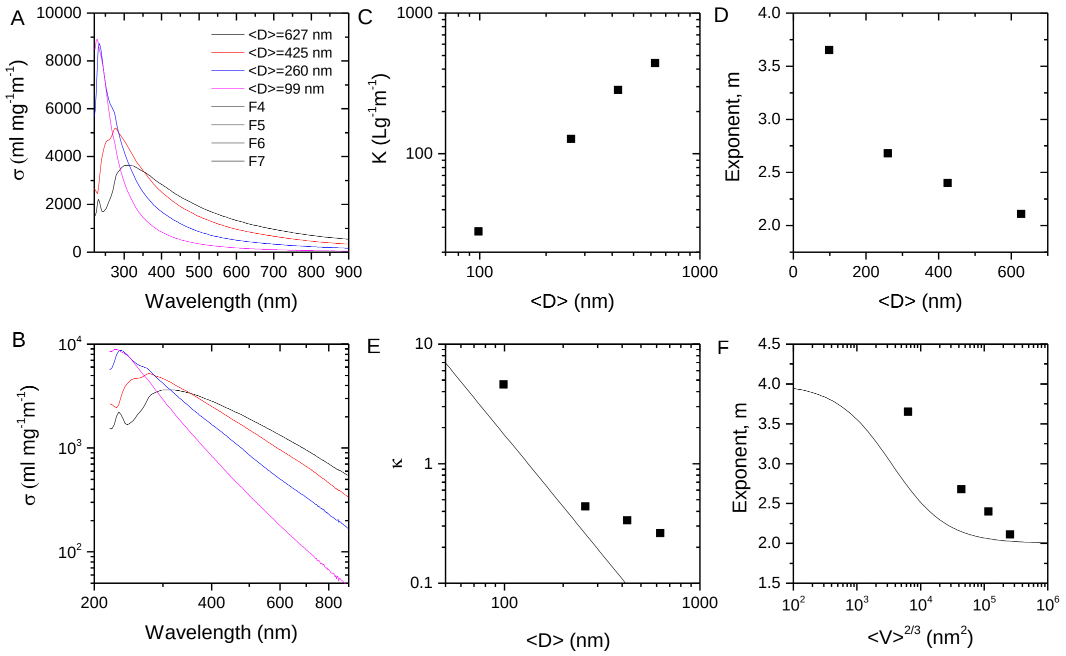


**Supplementary Figure 26: Scattering from nanospheres.** Samples of polystyrene nanospheres dispersed in water with different diameters (D) were purchased from microparticles GmbH (D-12489). Extinction, absorption and scattering measurements were made in the same way as for the platelets in the main text with scattering spectra shown in A-B. By fitting the long-wavelength power law region, K and m were extracted as shown in C-D. Using the methods described in the main text, we extracted κ which is plotted versus D in figure E. The exponent, m, was plotted versus $V^{2/3}$ in figure F, where v is the nanosphere volume. The fit lines found in the main text for nanosheets are included in figure E-F. We find the sphere data to sit relatively close to the platelet fit line for κ but well off the fit line for m.

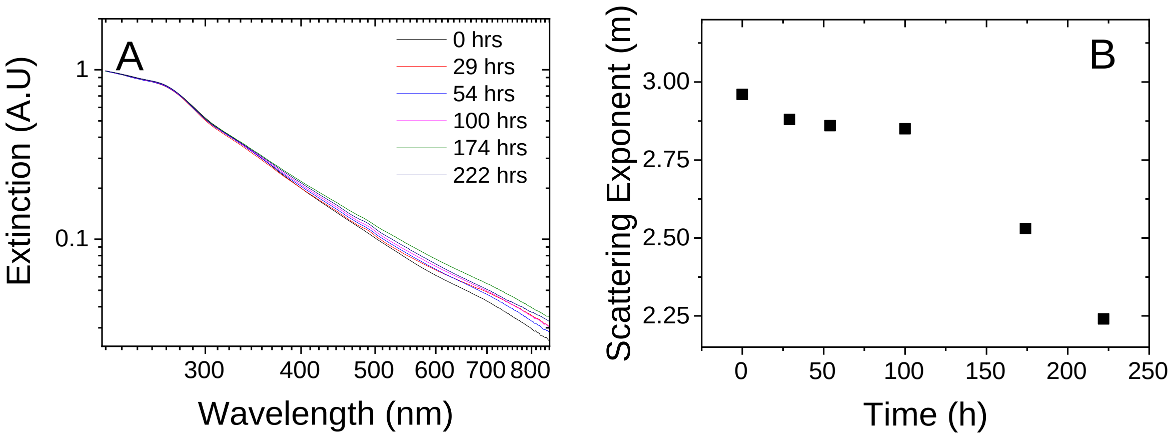


**Supplementary Figure 27: Reaggregation kinetics- time dependence m-Cu(OH)₂.** A) Normalised extinction spectra versus wavelength. B) Scattering exponent (m) of a copper hydroxide dispersion prepared as described in the methods section plotted against time after the sample was prepared. The scattering exponent stays reasonably constant for 100 h and then falls off as the nanosheets begin to reaggregate causing bigger clusters within the dispersion. Comparing to supplementary figure 28, this occurs later in $Cu(OH)_2$ compared to $Co(OH)_2$ suggesting that $Co(OH)_2$ is more prone to aggregation.

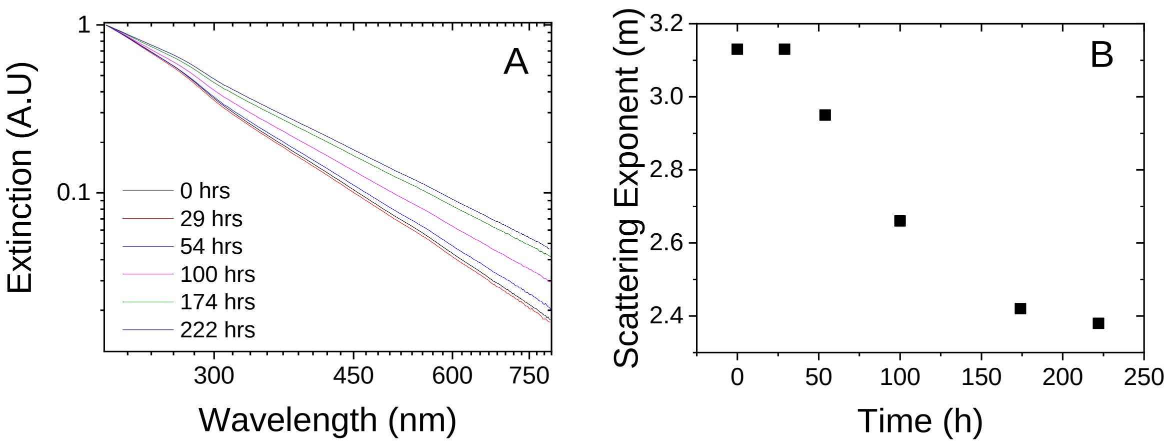


**Supplementary Figure 28: Reaggregation kinetics- time dependence m-Co(OH)₂.** A) Normalised extinction spectra versus wavelength. B) Scattering exponent (m) of a cobalt hydroxide dispersion prepared in analogy to the other hydroxides used in the study. The scattering exponent stays reasonably constant for 30 h and then falls off as the nanosheets begin to reaggregate. This occurs sooner than in $Cu(OH)_2$, compare supplementary figure S27.

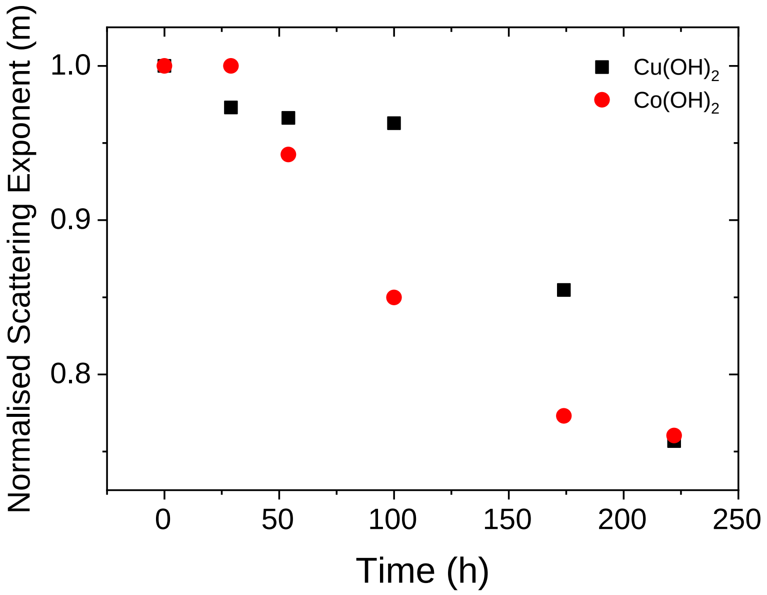


**Supplementary Figure 29: Reaggregation kinetics- comparison m-$Cu(OH)_2$ and m-$Co(OH)_2$.** Scattering exponent (m) of cobalt and copper hydroxide dispersions plotted as function of time to illustrate that reaggregation which is accompanied by a drop in the scattering exponent occurs sooner in $Co(OH)_2$ than $Cu(OH)_2$.

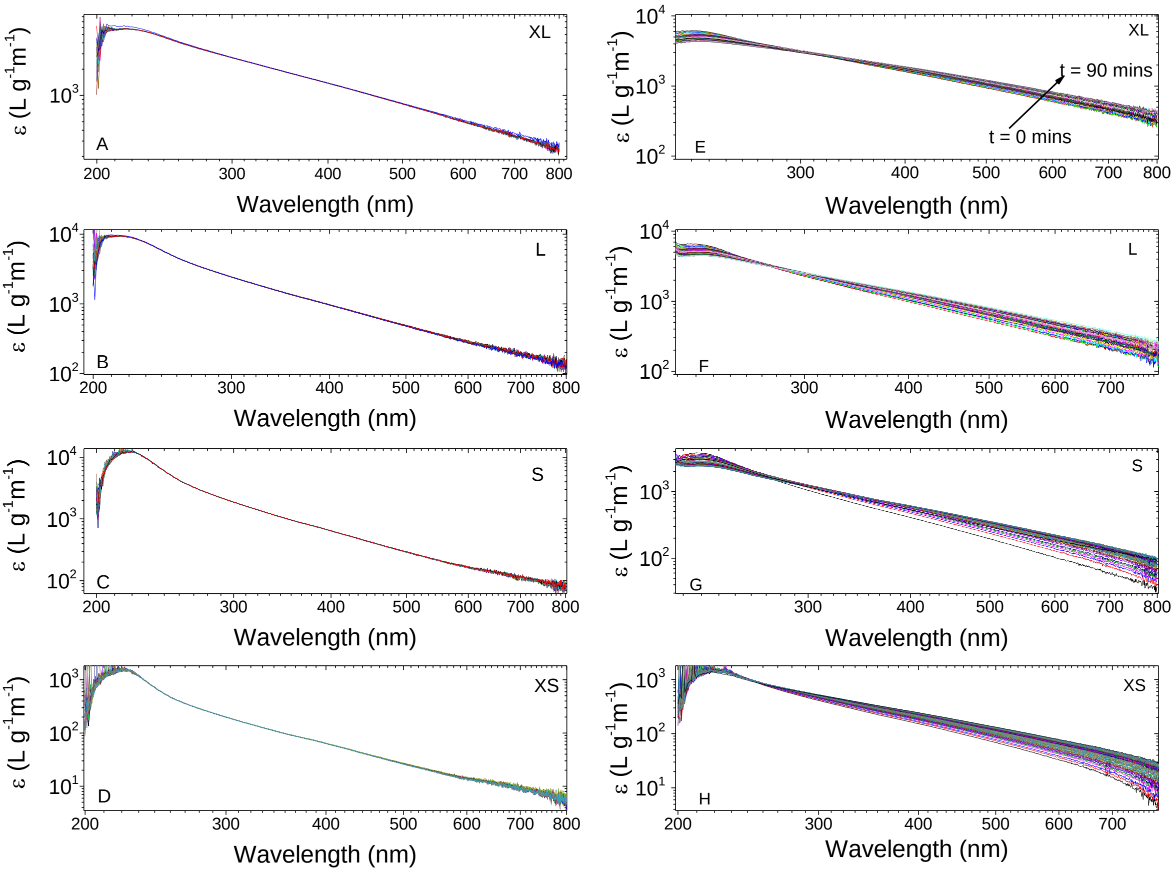


**Supplementary Figure 30: Size-dependent reaggregation kinetics after addition of salt-spectra.** To study the effect of nanosheet size on the aggregation, the aggregation was speed up by the addition of NaCl (see methods). $Ni(OH)_2$ was used as model substance. Extinction coefficient spectra of size-selected $Ni(OH)_2$ measured over 90 minutes with (A-D) no salt added and (E-H) salt added. It can be seen that when no salt is added that there is no reaggregation in $NiOH_2$ in this time frame, as the spectra in A-D are virtually unchanged. In contrast, when salt is added, there is a very large change in the shape of the spectra as shown E-H.

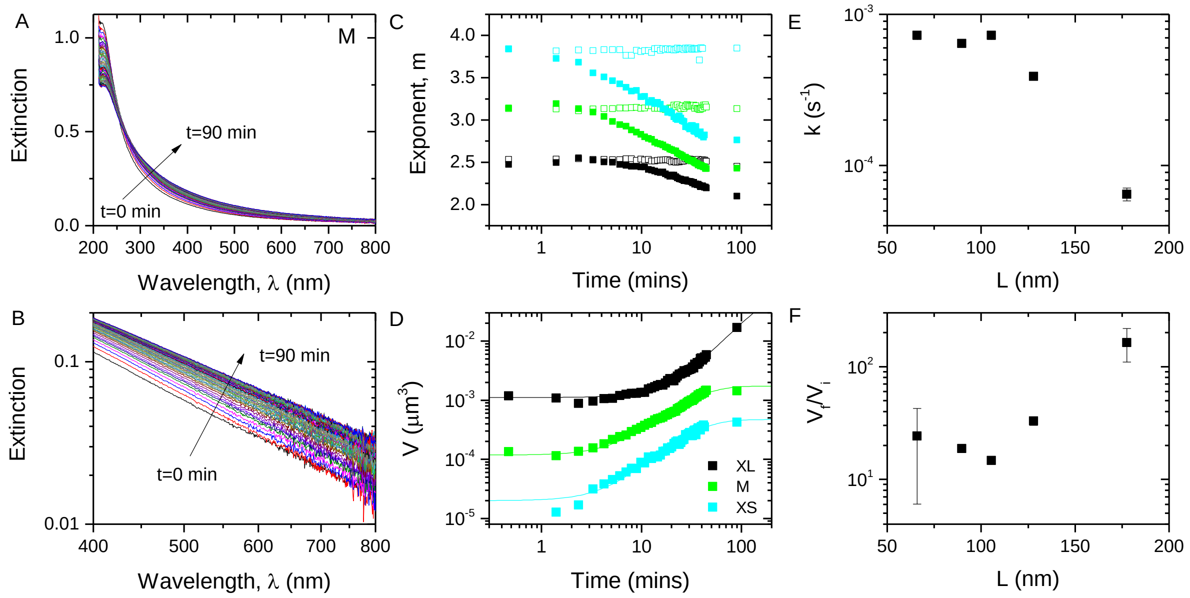


**Supplementary Figure 31: Size-dependent reaggregation kinetics after addition of salt-scattering exponent and volume.** Tracking aggregation using light scattering. A) Extinction spectra for dispersions of size-selected $Ni(OH)_2$ nanosheets (size M) for different times after the addition of NaCl to a concentration of 5 g/L (9 mM). B) Magnified version of the same spectra plotted on a log-log plot. C) Exponent, m, extracted from spectra such as those in B, plotted versus time after salt addition (all 10 g/L), for dispersions of size selected nanosheets from XL, M, XS fractions (L and S not shown to avoid clutter). The filled symbols represented dispersions to which salt had been added while the open symbols represent control samples to which no salt was added. D) Aggregate volume, extracted from m-data using equation 10, plotted versus time after salt addition. The lines are fits to the empirical equation $V = V_i + V_f\left(1 - e^{-kt}\right)^2$. The legend in D also represents the data in C. E-F) Rate constant (E) and ratio of final aggregate to initial nanosheet volume (F), as extracted from the fits to $V = V_i + V_f\left(1 - e^{-kt}\right)^2$, plotted versus the mean length of the nanosheets in each fraction (XL to XS). Error bars represent the error in the fit.

## Supplementary Table

**Supplementary Table 1: Optical constants.** Density ρ, solvent refractive index $n_0$ and nanosheet refractive index $n$. For all materials except GaS (IPA), the solvent was water.

| | $\rho$ (kg/m$^3$) | $n_0$ | $n$ |
|---|---|---|---|
| $Ni(OH)_2$ | 4100 | 1.33 | 1.759[3] |
| $Mg(OH)_2$ | 2340 | 1.33 | 1.567[3] |
| $Cu(OH)_2$ | 3370 | 1.33 | 1.760* |
| GaS | 3860 | 1.47 | 2.35[4] |
| BN | 2100 | 1.33 | 1.70[5] |
| Talc | 2750 | 1.33 | 1.57** |

* https://en.wikipedia.org/wiki/Spertiniite

** http://www.science.smith.edu/geosciences/petrology/Petrography/talc/talc.html

## Supplementary Note 1

### Discussion of Raman spectra

To confirm that the composition of the nanosheets was not changed on exfoliation, a subset of the dispersions (XL and S) were characterised by Raman spectroscopy. For this purpose, the hydroxide and talc dispersions were centrifuged twice at 10000 *g* and redispersed with water to remove surfactant and drop cast on glass substrates covered with alumina foil (to supress background signal). Surfactant removal and suppression of background signal is crucial in this case due to the mostly non-resonant nature of the excitation at 532 nm. The GaS was directly dropcast on the substrates from isopropanol. The mean of ~ 100 spectra was baseline corrected and is displayed in supplementary figure 1. The characteristic nanosheet lattice vibrations are observed with no significant changes in the Raman spectra of the XL and S dispersions except for some variations in relative peak intensity ratios. This confirms that the structure of the nanosheets was not altered on exfoliation and size selection.

The Raman spectra of talc (Figure S1A) show characteristic lattice and molecular vibrations in agreement with literature.[6-8] Peaks were assigned according to [8]. At ~ 194 $cm^{-1}$, the A1g lattice vibration associated with the $MgO_6$ octahedron is observed. In addition to weak signatures, two pronounced molecular vibrations of $SiO_4$ are discerned at ~362 cm-1 and ~676 $cm^{-1}$. The Raman spectra of the exfoliated BN and GaS (Figure S1B and C) are in agreement with published work on exfoliated nanosheets and show the expected lattice vinrations.[1,9]

The Raman spectra of the hydroxides under study are shown in figure S1D-E). In addition to the lattice vibrations at 200-550 cm-1, the OH molecular OH stretching mode is clearly visible at 3500-3700 $cm^{-1}$. This mode is the dominant peak in $Mg(OH)_2$ and $Ni(OH)_2$, but a bit weaker in $Cu(OH)_2$, as the latter is slightly resonant at the excitation wavelength. In $Cu(OH)_2$ sidebands are observed at 3200-3500 possibly due to surface and disorder O-H as frequently observed in $Ni(OH)_2$.[10,11] The lattice vibrations of $Mg(OH)_2$ and $Ni(OH)_2$ were assigned according to ref. [10-12]

In this case, the dominant lattice vibrations are $E_g$ and $A_{1g}$ phonons, with the $A_{2u}$ vibration also discernible in $Ni(OH)_2$. For $Cu(OH)_2$, no assignment to lattice vibrations could be found in literature and the peaks were therefore tentatively assigned to $E_g$, $A_{1g}$ and $A_{2u}$ in analogy to other brucite-type hydroxides.[13] The vibrations at < 300 $cm^{-1}$ in $Cu(OH)_2$ and $Mg(OH)_2$ could not be assigned to phonons, but it should be noted that this peak was previously observed for $Cu(OH)_2$ nanowires[14] and is found in the mineral spertiniite.[15]

**Supplementary Note 2~~3~~**

## Scattering parameter conversion and approximation for large disks

*Scattering parameter conversion and the approximate use of* $(n/n_0 - 1)^2$

In this paper we discuss scattering coefficients, σ, as the most appropriate parameter to apply to data. More often in scattering, the scattering cross section, $\sigma_{CS}$ is used. These parameters are related via $\sigma = \sigma_{CS}/(\rho V)_{particle}$ where ρ and V are the scattering particle density and volume. Alternatively, the scattering efficiency, $Q$, is used. This is defined as the ratio of the scattering cross section to the geometric cross section i.e $Q = \sigma_{CS}/(\pi D^2/4)$ for a sphere. In addition, in some works (particularly the vdH textbook), the parameter x is often used, where $x = kD/2 = \pi D/\lambda$. Finally, the ratio of particle to solvent refractive index is often written in the literature as $m = n/n_0$ (not to be confused with the symbol for scattering exponent used in this paper).

Van der Hulst[16] (1981 edition, p70) writes the standard result for scattering by small spheres (Rayleigh scattering) as

$$Q = \frac{8}{3} x^4 \left| \frac{m^2 - 1}{m^2 + 2} \right|^2$$

Which is equivalent to equation 3 in the main text.

In addition, van de Hulst quotes a result which is approximate for small spheres (1981 edition, p90):

$$Q = \frac{32}{27} x^4 (m-1)^2$$

This is equivalent to applying the approximation

$$\left| \frac{m^2 - 1}{m^2 + 2} \right|^2 \approx \frac{4}{9}(m-1)^2$$

This approximation is accurate to better than 80% (i.e. 20% deviation) so long as m<1.5.

*Approximations for large disks*

The expanded van der Hulst approximation for large spheres (equation 7 main text) can be written in the alternative form (using van der Hulst's notation):

$$Q=\frac{1}{2}\rho^2=\frac{1}{2}\left[2x(m-1)\right]^2$$

In his textbook (1981 edition), vdH shows that for both disks and cylinders oriented perpendicular to the incident beam, Q is given by $Q=\rho^2$ and $Q=\frac{2}{3}\rho^2$ respectively so long as x>>1 where $x=\pi D/\lambda$ with D referring to both disk and cylinder diameter. (See 1981 edition, p97).

This means that for all three geometries (at least in certain orientations) $\sigma\propto Q\propto\left[2x(m-1)\right]^2\propto(m-1)^2\lambda^{-2}$. However, we not that we cannot use this result as it stands as it is derived for one specific geometry and does not specifically apply to randomly orientated nanosheets as would exist in a dispersion.

**Supplementary Note 3:**

**Fitting of scattering exponent versus nanosheet dimension**

In order to find a mastercurve for the scattering exponent, *m*, we used trial and error, plotting *m* versus various parameters related to the mean dimensions of the nanosheets (i.e. mean length, width and thickness - <L>, <w>, <t>), see supplementary figure 25. Of these the mean planar nanosheet area, mean nanosheet volume and (mean nanosheet volume)^2/3 (all bottom row) showed reasonable master-curve-like behaviour. N.B. the (mean nanosheet volume)^2/3 [bottom right] represents a characteristic area associated with the nanosheets and reflects the mean projected nanosheet area intersecting the beam. To assess the best candidate for mastercurve-like behaviour, we fitted the data sets in the bottom row to a generic function: $m = a + \frac{(4-a)}{1+x/b}$ where x represents the dimensional parameter. Fitting yielded the following results

| | a | b | $R^2$ |
|---|---|---|---|
| Mean planar NS area | 2.36±0.15 | 11920±3230 $nm^2$ | 0.73 |
| Mean NS volume | 2.41±0.1 | 91510±20870 $nm^3$ | 0.78 |
| Mean NS projected area | 2.07±0.15 | 3006±664 $nm^2$ | 0.80 |

Based on these results (i.e. the highest value of $R^2$), we take the mean NS projected area as the best dimensional parameter to form a mastercurve with m. In addition, we note that the parameter a represents the value of m when the x parameter is very large. For the m versus mean NS projected area fit, we find a=2 within error. This implies very large nanosheets to behave in a manner similar to the vdH approximation (i.e. m=2). Thus, in the main text, we have fixed a=2.

It should be noted that we have defined the nanosheet volume as half the product of length, width and thickness. The factor of 2 comes from the fact that liquid-exfoliated nanosheets tend to be irregularly shaped with planar areas smaller than the product of length and width. Previous work showed the mean planar area to be approximately half the product of mean length and width.[17]

In addition, it should be pointed out that we have written some of the dimensional parameters in a manner which is not strictly correct e.g. <V>=<L><w><t>/2 rather than the

correct version: <V>=<Lwt>/2. The reason for this is to allow the scattering exponent mastercurves to be used as size metrics. For example, as described in the main text, once *m* is obtained from fit, equation 10 can be used to estimate the mean nanosheet volume $\langle V\rangle$. These data can be combined to estimate the mean nanosheet thickness, $\langle t\rangle$, using $\langle V\rangle \approx \langle L\rangle\langle w\rangle\langle t\rangle/2$. Most liquid exfoliated nanosheets have $\langle W\rangle \sim \langle L\rangle/1.5$ (figure 3G). This allows us to estimate mean nanosheet thickness using $\langle t\rangle \sim 3\langle V\rangle/\langle L\rangle^2$ if <L> is known (e.g. from TEM).

**Supplementary Note 4:**

**Scattering metrics for size estimation**

In principle, equation 9 can be used to estimate nanosheet size information from scattering coefficient spectra. Equations 9 can be used to fit the long-wavelength, power-law part of the scattering spectrum (N.B. σ [L/g/m] should be plotted versus wavelength in meters for fitting). Then, from the fit parameters, assuming $\rho$, *n* and $n_0$ are known and taking $L_0$ =142 nm, one can estimate $\langle L\rangle$. In addition, once *m* is obtained from the fit, equation 10 can be used to estimate the mean nanosheet volume $\langle V\rangle$. These data can be combined to estimate the mean nanosheet thickness, $\langle t\rangle$, using $\langle V\rangle \approx \langle L\rangle\langle w\rangle\langle t\rangle/2$. Most liquid-exfoliated nanosheets have $\langle W\rangle \sim \langle L\rangle/1.5$ (figure 3G). This allows us to estimate mean nanosheet thickness using $\langle t\rangle \sim 3\langle V\rangle/\langle L\rangle^2$.

In principle the procedure above should be applied to the scattering coefficient spectrum. However, for most wide bandgap semiconductors or insulators, the scattering and extinction coefficient spectra are virtually identical in the non-resonant regime (figure 4). This allows size information to be obtained from the long-wavelength part of the extinction coefficient spectrum. In addition, if the concentration is not known such that only extinction spectra rather than extinction coefficient spectra are available, the mean nanosheet volume can still be estimated from the scattering exponent using equation 10.

We can use this last point to illustrate the utility of these results by using the scattering exponent to probe nanosheet aggregation. For example, we can track a dispersion as function of time to gain insights whether a material aggregates more or less quickly. This is exemplarily shown for $Co(OH)_2$ and $Cu(OH)_2$ in the SI (Figure S27-29). Probably of even greater interest,

aggregation kinetics can be studied for nanosheets of different dimensions. It is well known that the addition of salt to a surfactant-stabilised colloidal dispersion results in a rapid destabilisation followed by aggregation and sedimentation.[25,26] We prepared a surfactant-stabilised dispersion of $Ni(OH)_2$ nanosheets which was size-selected into five fractions labelled XL to XS as before. To each fraction we added NaCl to a concentration of 5 g/L (~9 mM). We then measured the extinction spectrum for each fraction at 1 min intervals over a period of 90 minutes. We also measured spectra at similar intervals for control dispersions to which no salt had been added (Figure S30).

While no changes were seen for the extinction spectra of the control samples, the salted dispersions showed subtle but consistent changes (figure 31A) which were dominated by a steady reduction of the scattering exponent with time (figure 31B and figure S32). For each control (open symbols) and salted (filled symbols) dispersion, we extracted values for the scattering exponent, *m*, which are plotted versus time in figure S31C. While the control dispersions show no change in *m* over 90 minutes, the salted samples showed a steady decay in *m* over time with a noticeable difference observable after only a few minutes.

With reference to equation 10, this decrease in *m* is consistent with a steady growth in the volume of the suspended particles as nanosheets aggregate to give larger structures. Assuming the resultant aggregates are platelet-like, we can use equation 10 to convert the data in figure S31C into aggregate volume as a function of time as shown in figure S31D. This data shows a steady increase in aggregate volume over time with some evidence of saturation at long times.

We found the time dependence of the aggregate volume to be consistent with an empirical stretched-exponential-like function:

$$V = V_i + V_f \left(1 - e^{-kt}\right)^2$$

where $Vi$ is the initial nanosheet volume, $V_f$ is the final aggregate volume and $k$ can be thought of as a rate constant. Fitting this data to the experimental curves allows us to obtain $k$ as well as the ratio $V_f/V_0$ which can be thought of as the number of nanosheets per aggregate at saturation. The resultant time constants are plotted versus the mean (initial) nanosheet length for each fraction in figure 31E. This shows the time constant to fall significantly with nanosheet size for $\langle L \rangle$ >100 nm. This may be reflective of the reduction in nanosheet diffusion coefficient for larger sheets. However, $V_f/V_0$ tends to increase with increasing nanosheet size as shown in

figure 31F. While the saturated aggregates consist of tens of nanosheets for the smaller sizes, for the larger sizes, the final aggregates may contain hundreds of aggregates, illustrating the greater propensity of larger sheets toward aggregation.

**Aggregation Kinetics Methods**: NaCl was added to surfactant-stabilised dispersions of size-selected $Ni(OH)_2$ nanosheets (5 fractions produced as described above, SC concentration 9 g/L, nanosheet conc  0.05 g/L) to yield a NaCl concentrations of 5 g/L (~9 mM). Extinction spectra for each fraction at 1 min intervals were measured over a period of 90 minutes.

## Supplementary References


1. Griffin, A. *et al.* Spectroscopic Size and Thickness Metrics for Liquid-Exfoliated h-BN. *Chem. Mater.* **30**, 1998-2005, (2018).
2. Backes, C. *et al.* Production of Highly Monolayer Enriched Dispersions of Liquid-Exfoliated Nanosheets by Liquid Cascade Centrifugation. *ACS Nano* **10** 1589-1601, (2016).
3. Shannon, R. C., Lafuente, B., Shannon, R. D., Downs, R. T. & Fischer, R. X. Refractive indices of minerals and synthetic compounds. *Am. Mineral.* **102**, 1906-1914, (2017).
4. Adachi, S. *The Handbook on Optical Constants of Semiconductors*. (World Scientific, 2012).
5. Stenzel, O. *et al. Phys. Stat. Sol. (a)* **158** 281-287., (1996).
6. Rosasco, G. J. & Blaha, J. J. Raman Microprobe Spectra and Vibrational Mode Assignments of Talc. *Appl. Spectrosc.* **34**, 140-144, (1980).
7. Alian, W., J., F. J. & L., J. B. Understanding the Raman spectral features of phyllosilicates. *J. Raman Spectrosc.* **46**, 829-845, (2015).
8. Loh, E. Optical vibrations in sheet silicates. *Journal of Physics C: Solid State Physics* **6**, 1091, (1973).
9. Harvey, A. *et al.* Preparation of Gallium Sulfide Nanosheets by Liquid Exfoliation and Their Application As Hydrogen Evolution Catalysts. *Chem Mater* **27**, 3483-3493, (2015).
10. Hall, D. S., Lockwood, D. J., Poirier, S., Bock, C. & MacDougall, B. R. Raman and Infrared Spectroscopy of α and β Phases of Thin Nickel Hydroxide Films Electrochemically Formed on Nickel. *The Journal of Physical Chemistry A* **116**, 6771-6784, (2012).
11. Hall, D. S., Lockwood, D. J., Bock, C. & MacDougall, B. R. Nickel hydroxides and related materials: a review of their structures, synthesis and properties. *Proceedings of the Royal Society A* **471**, 20140792, (2014).
12. Dawson, P., Hadfield, C. D. & Wilkinson, G. R. The polarized infra-red and Raman spectra of Mg(OH)2 and Ca(OH)2. *J. Phys. Chem. Solids* **34**, 1217-1225, (1973).
13. Lutz, H. D., Möller, H. & Schmidt, M. Lattice vibration spectra. Part LXXXII. Brucite-type hydroxides M(OH)2 (M = Ca, Mn, Co, Fe, Cd) — IR and Raman spectra, neutron diffraction of Fe(OH)2. *J. Mol. Struct.* **328**, 121-132, (1994).
14. Wang, W. *et al.* Raman optical properties of Cu(OH)2 nanowires. *Mater. Lett.* **63**, 2432-2434, (2009).
15. Project, R. *Spectral data base for minerals*, <http://rruff.info/spertiniite/chem=Cu/display=default/> (
16. van de Hulst, H. C. *Light Scattering by Small Particles*. (Courier Corporation, 1981).
17. Ferguson, A., Caffrey, I. T., Backes, C., Coleman, J. N. & Bergin, S. D. Differentiating Defect and Basal Plane Contributions to the Surface Energy of Graphite Using Inverse Gas Chromatography. *Chem. Mater.* **28**, 6355-6366, (2016).
18. Griffin, A. *et al.* Spectroscopic Size and Thickness Metrics for Liquid-Exfoliated h-BN. *Chem Mater* **30**, 1998-2005, (2018).
19. Backes, C. *et al.* Spectroscopic metrics allow in situ measurement of mean size and thickness of liquid-exfoliated few-layer graphene nanosheets. *Nanoscale* **8**, 4311-4323, (2016).
20. Ueberricke, L., Coleman, J. N. & Backes, C. Robustness of Size Selection and Spectroscopic Size, Thickness and Monolayer Metrics of Liquid-Exfoliated WS2. *Physica Status Solidi B-Basic Solid State Physics* **254**, (2017).
21. Backes, C. *et al.* Edge and confinement effects allow in situ measurement of size and thickness of liquid-exfoliated nanosheets. *Nat Commun* **5**, (2014).
22. Harvey, A. *et al.* Production of Ni(OH)(2) nanosheets by liquid phase exfoliation: from optical properties to electrochemical applications. *Journal of Materials Chemistry A* **4**, 11046-11059, (2016).
23. Harvey, A. *et al.* Exploring the versatility of liquid phase exfoliation: producing 2D nanosheets from talcum powder, cat litter and beach sand. *2D Materials* **4**, 025054, (2017).
24. McAteer, D. *et al.* Liquid Exfoliated Co(OH)(2) Nanosheets as Low-Cost, Yet High-Performance, Catalysts for the Oxygen Evolution Reaction. *Adv. Energy Mater.* **8**, (2018).

25 Chen, K. L. & Elimelech, M. Aggregation and deposition kinetics of fullerene (C-60) nanoparticles. *Langmuir* **22**, 10994-11001, (2006).

26 McDonald, T. J., Engtrakul, C., Jones, M., Rumbles, G. & Heben, M. J. Kinetics of PL Quenching during Single-Walled Carbon Nanotube Rebundling and Diameter-Dependent Surfactant Interactions. *The Journal of Physical Chemistry B* **110**, 25339-25346, (2006).
